# Introduction to nanomaterials in catalysis and sensing applications

**DOI:** 10.1039/d5na90028j

**Published:** 2025-05-29

**Authors:** Thanh-Danh Nguyen, Dinh Quang Khieu, Nguyen Hoang Tuan, Mita Dasog

**Affiliations:** a Institute of Chemical Technology, Vietnam Academy of Science and Technology Vietnam; b Hue University Vietnam; c Jeonbuk National University Republic of Korea; d Dalhousie University Canada

## Abstract

Thanh-Danh Nguyen, Dinh Quang Khieu, Nguyen Hoang Tuan and Mita Dasog introduce the *Nanoscale Advances* themed collection on nanomaterials for catalysis and sensing.
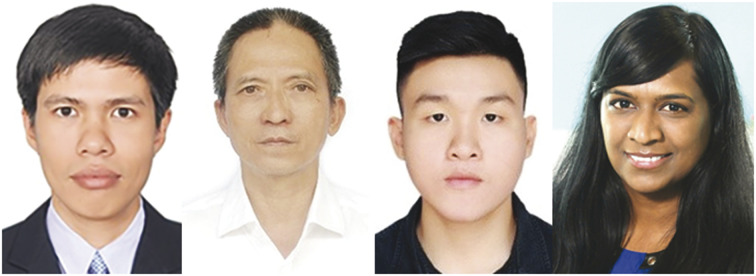

Nanomaterials have revolutionized numerous fields, including biology, environmental science, agriculture, and healthcare. In catalysis, they offer enhanced selectivity, recyclability, and efficiency, while their unique properties have driven advances in sensing technologies for environmental monitoring and biomedical diagnostics. This themed collection highlights recent breakthroughs in these domains, demonstrating how nanomaterials bridge the gap between fundamental research and practical applications.

Tamtam *et al.* (https://doi.org/10.1039/D5NA00019J) synthesized bimetallic Cu/Co-MOFs using three synthetic approaches, demonstrating exceptional stability in asymmetric and symmetric coin cell devices. Complementing this, Weheabby *et al.* (https://doi.org/10.1039/D4NA00919C) advanced sensing technologies by developing an electrochemical sensor for methyl parathion detection, utilizing AgNPs@GO/IL@SPCE, achieving a detection limit of 0.009 μmol L^−1^ with excellent selectivity and stability over 60 days.

The role of nanomaterials in catalysis is further underscored by Nguyen *et al.* (https://doi.org/10.1039/D4NA00979G), who reported AuNP-embedded magnetic nanocomposites (AuNPs/Fe_3_O_4_@GluN/Alg) for nitrophenol reduction, exhibiting high catalytic efficiency and recyclability. Similarly, Tran *et al.* (https://doi.org/10.1039/D4NA00707G) explored PANI nanoparticles for visible-light-driven dye degradation, achieving 97.09% methylene blue removal with sustained photostability, highlighting the potential of nanomaterials in environmental remediation.

Expanding into sustainable energy applications, Nguyen *et al.* (https://doi.org/10.1039/D4NA00892H) developed a defect-rich CeO_*x*_/β-Ni(OH)_2_ electrocatalyst for glucose oxidation-assisted hydrogen production, delivering nearly 100% faradaic efficiency. This aligns with the work of Conlin *et al.* (https://doi.org/10.1039/D4NA00854E), who investigated the stability of ALD-synthesized TiO_2_ and ZnO films under CO_2_ plasma exposure, confirming their robustness for catalytic applications in carbon capture and conversion. Further advancing catalytic efficiency, Ta and Nhiem (https://doi.org/10.1039/D4NA00947A) designed Au single-atom catalysts on TiO_2_ for photocatalytic methane oxidation, achieving a hydrogen production rate of 2190 μmol g^−1^ and 58% selectivity *via* a methyl radical pathway, offering a promising route for methane valorization.

This collection highlights the transformative role of nanomaterials in catalysis and sensing applications, showcasing their potential to address global challenges. By bridging disciplines and fostering innovation, we hope these contributions will inspire further collaboration and accelerate the translation of nanotechnology from the laboratory to real-world solutions. As Guest Editors, we sincerely appreciate the invaluable support of the *Nanoscale Advances* editorial team, the authors for their outstanding contributions, and the reviewers for their insightful evaluations that have helped shape this impactful compilation. In particular, we extend our gratitude to the contributors to the Second Green Chemistry Conference in the Central and Highlands Region, Vietnam, held at Hue University on November 30, 2024, for their valuable insights and engagement.

